# Molecular detection of *Leishmania infantum* in rats and sand flies in the urban sewers of Barcelona, Spain

**DOI:** 10.1186/s13071-022-05309-4

**Published:** 2022-06-16

**Authors:** María Teresa Galán-Puchades, Jennifer Solano, Gloria González, Antonio Osuna, Jordi Pascual, Rubén Bueno-Marí, Sandra Franco, Víctor Peracho, Tomás Montalvo, Màrius V. Fuentes

**Affiliations:** 1 Parasite and Health Research Group, Department of Pharmacy, Pharmaceutical Technology and Parasitology, Faculty of Pharmacy, University of Valencia, 46100 Burjassot, Valencia Spain; 2 Molecular Biochemistry and Parasitology Research Group, Department of Parasitology, Institute of Biotechnology, Faculty of Sciences, University of Granada, 18071 Granada, Spain; 3 Pest Surveillance and Control, Agència de Salut Pública de Barcelona (ASPB), 08023 Barcelona, Spain; 4 Department of Research and Development, Laboratorios Lokímica, 46980 Paterna, Valencia Spain; 5 Biomedical Research Center Network for Epidemiology and Public Health CIBERESP Epidemiology and Public Health, 08023 Barcelona, Spain

**Keywords:** *Leishmania infantum*, *Phlebotomus perniciosus*, *Rattus norvegicus*, Barcelona sewer system, Underground leishmaniasis

## Abstract

**Background:**

Classically, dogs have been considered to be the only reservoir of leishmaniasis in urban areas. However, in a previous study, we found a 33.3% prevalence of *Leishmania infantum* in the spleens of Norway rats (*Rattus norvegicus*) sampled in the underground sewer system of the city of Barcelona (Spain). The aim of the present study was to verify, using molecular methods, the potential reservoir role of these rats in the same sewer system.

**Methods:**

A sensitive real-time PCR (qPCR) assay, DNA sequencing and phylogenetic analysis were carried out to identify and quantify the presence of *L. infantum* DNA in sand fly individuals captured in the same underground sewer system of Barcelona as in our previous study and in the spleens and ears of rats captured in the same sewer system.

**Results:**

*Leishmania infantum* DNA was found in 14 of the 27 (51.9%) sand flies identified as *Phlebotomus perniciosus*, and 10 of the 24 (41.7%) rats studied were infected. *Leishmania infantum* was found in the spleens (70%) and in the ears (40%) of the infected rats. Quantitative results revealed the presence of high loads of *L. infantum* in the rats studied (> 3 × 10^6^ parasites/g ear tissue) and among the sand flies (> 34 × 10^6^ parasites in 1 individual).

**Conclusions:**

The molecular methods used in this study demonstrated a high prevalence of *L. infantum* in the underground sewer populations of both* R. norvegicus* and* P. perniciosus*. These results suggest that sewer rats, in addition to dogs, are likely to act as reservoirs of leishmaniasis in cities, where sewer systems seem to offer the ideal scenario for the transmission of leishmaniasis. Therefore, to achieve the WHO 2030 target on the elimination of leishmaniasis as a public health problem successfully, an efficient control strategy against leishmaniasis in rats and sand flies should be implemented, particularly in the sewer systems of urban areas of endemic countries.

**Graphical Abstract:**

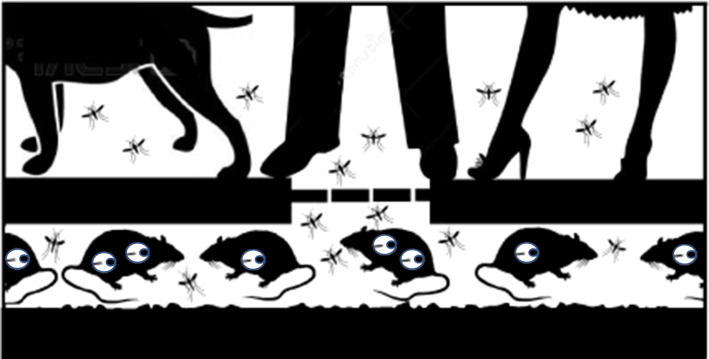

**Supplementary Information:**

The online version contains supplementary material available at 10.1186/s13071-022-05309-4.

## Background

The road map for neglected tropical diseases (NTDs) 2021–2030, elaborated by the WHO and endorsed by the Seventy-third World Health Assembly in November 2020, sets global targets and milestones to prevent, control, eliminate or eradicate 20 diseases. These 20 diseases and disease groups, classified as NTDs, have one feature in common: their devastating impact on impoverished communities [1]. Leishmaniasis, a vector-borne disease transmitted by the bite of phlebotomine females, is caused by 20 *Phlebotomus* species pathogenic to humans, and the disease ranks among the top ten NTDs worldwide [2]. About 350 million people are at risk of acquiring the disease in 97 endemic countries. In terms of disease burden, in 2019, 277,224 new cases of cutaneous leishmaniasis (CL) and 13,814 of visceral leishmaniasis (VL) (including 491 deaths) were reported in endemic countries [1]. In the Mediterranean basin, of the four *Leishmania* species present (*Leishmania infantum, L. major*, *L. tropica* and *L. donovani*), *L. infantum* is predominant and the causative agent of CL, VL and mucocutaneous leishmaniasis [3]. A dozen *Phlebotomus* species have been implicated in the transmission of Mediterranean *L. infantum*, of which eight have been implicated as vectors, i.e. *Phlebotomus ariasi*, *P. balcanicus*, *P. kandelakii*, *P. langeroni*, *P. neglectus*, *P. perfiliewi*, *P. perniciosus* and *P. tobbi* [4].

In the context of animal reservoirs of *L. infantum,* dogs are classically considered to be the only vertebrate reservoir of leishmaniasis in urban areas in Mediterranean countries. Consequently, dogs are the only target for disease surveillance and control [5, 6].

Spain is one of the endemic countries of leishmaniasis in the Mediterranean basin [7], with *P. perniciosus* and *P. ariasi* being the main vectors of *L. infantum,* which is endemic in the Iberian Peninsula [4]. Studies in different areas of Spain have reported seroprevalences of canine leishmaniasis (CanL) in dogs varying from 0 to 57.1% [8]. Specifically in Barcelona province, the seroprevalence of CanL has been reported to range from 14.5 to 16.7% [8].

As part of the multifocal project “BCN Rats” program developed in Barcelona (Spain), we investigated and quantified the presence of *L. infantum* in the spleens of an urban population of the Norway rat (*Rattus norvegicus*), in a sampled population of 98 individuals, using a highly sensitive real-time PCR (qPCR) method for *Leishmania* DNA detection. Only one out of 14 (7.1%) rats captured in parks tested positive for *L. infantum.* However, the prevalence of *Leishmania* DNA in rats captured from the underground sewage system was 33.3% (28/84 rats) [9]. This was the first time that rats trapped in sewers were analyzed from a parasitological point of view. Thus, when only sewer rats are considered—and not those living above ground—a 0.13 rat-per-person scenario is suspected in Barcelona [10]. Based on this ratio, there could be more than 72,000 *L. infantum*-positive rats living in the underground sewers in Barcelona, a city with 1,664,182 inhabitants in 2020, representing a major public health concern as a potential reservoir.

The criteria to define a reservoir in the case of leishmaniasis were established by WHO in 2010 [11]. Specifically, the reservoir must be abundant and long lived; an intense host-sand fly contact is necessary; *Leishmania* prevalence should be > 20%; the infection should be non-pathogenic and persistent; and the parasite should be available in blood or skin. Norway rats meet most of these criteria [12]. However, the host-sand fly contact, which can be proved by collecting infected vectors in sewers, and the presence of *L. infantum* in the skin of the rats, were not proved in our previous study.

In the present article, we report evidence showing high levels of *L. infantum* in sand flies as well as in the skin of the rats by qPCR, in specimens captured in the sewer system of Barcelona. We also demonstrate the presence of high *L. infantum* loads among sand fly and rat individuals.

## Methods

**Fig. 1:**
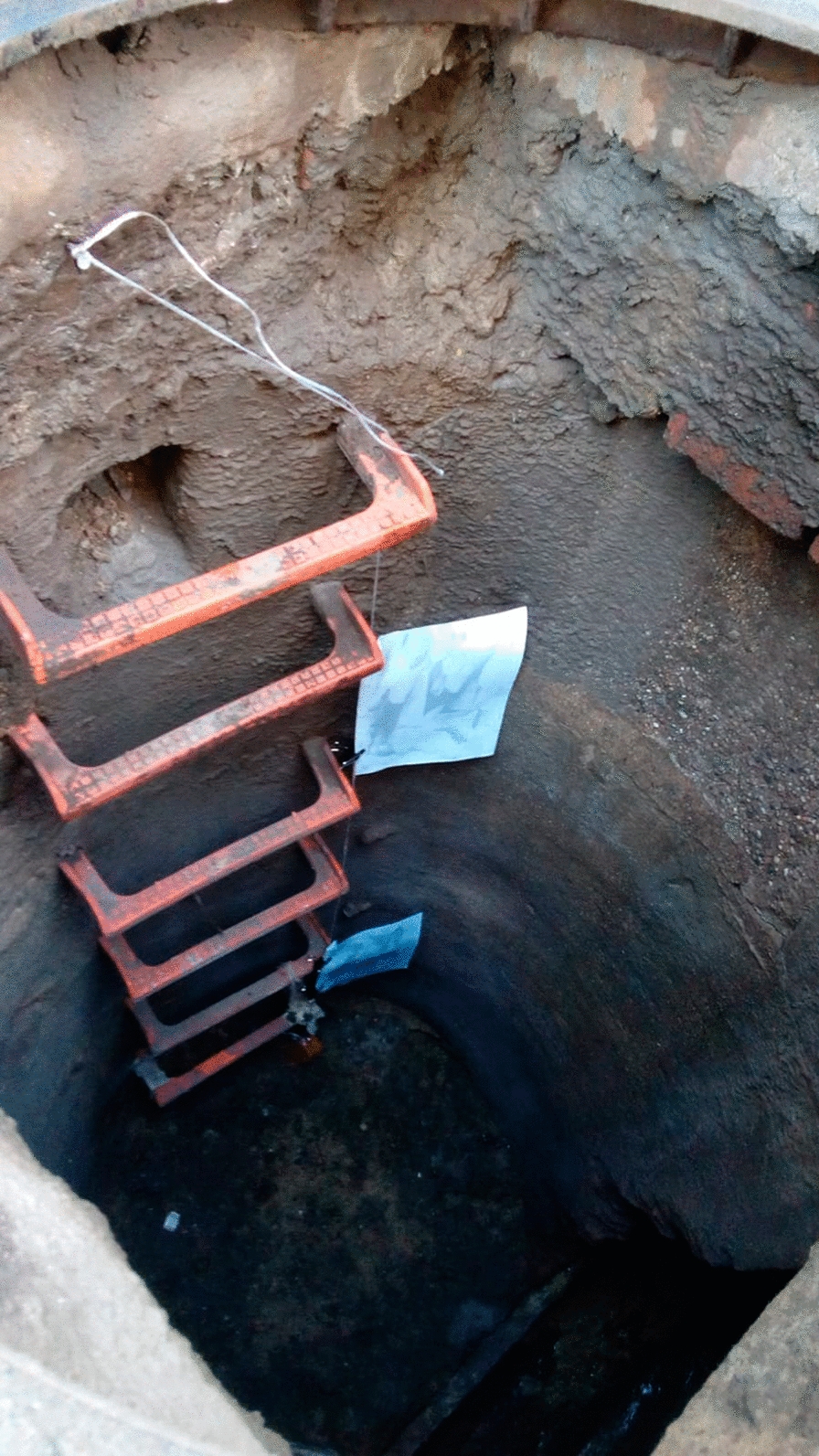
Sticky traps placed in the Barcelona sewer system

A total of 609 sand fly individuals were captured with sticky traps in 66 out of the 167 different sections prospected in the underground sewer system of Barcelona (unpublished data). Sampling was mainly carried out at those points in the sewer system where *Leishmania* parasite prevalences were highest according to our previous study [9]. A total of 25 points was sampled on a weekly basis in August/September 2018. A4-sheets impregnated with castor oil [13] were placed in each sewer section, one at the top of the section and the other at the bottom, in order to capture the maximum number of specimens (Fig. 1). Once the traps had been collected, they were kept at 4º C until treated to separate and identify the phlebotomine sand flies. Individuals separated from the traps by means of a paintbrush were placed in absolute ethanol to remove the castor oil and then stored in 70% ethanol until their subsequent assembly and identification. Twenty-seven randomly selected females of the trapped sand flies were identified by means of morphological keys [14–16].

New individuals of *R. norvegicus* were also captured with snap traps in the sewage system as part of the rodent surveillance and control program in Barcelona.

For the molecular detection of *Leishmania* spp. in the female sand flies, genomic DNA was extracted from individual phlebotomines by incubation overnight in 500 µl of TESCa + pK buffer (30 mM Tris–HCl, pH 8, 10 mM EDTA, 1% SDS, 5 mM CaCl_2_ and 0.42 µg/µl proteinase K). After incubation, an equal volume of a mixture of chloroform:isoamyl alcohol (24:1) was added to the incubation medium, followed by a vortexing step and centrifugation at room temperature for 5 min at 14,000 rpm. The top aqueous layer was transferred to a different tube and the DNA was precipitated in a 2.5 volume of absolute ethanol. All pellets were dissolved in 10 μl of nuclease-free water [17]. The DNA concentration and the absorbance ratio at 260 and 280 nm (A260/280 ratio) were evaluated using a NanoDrop OneC spectrophotometer (Thermo Fisher Scientific, Waltham, MA, USA). DNA extraction quality was evaluated with an internal control PCR, using the primers LCO1490 (5ʹ-GGTCAACAAATCATAAAGATATTGG-3ʹ) and HCO2198 (5ʹ-TAAACTTCAGGGTGACCAAAAAATCA-3ʹ), which amplify a cytochrome* c* oxidase subunit 1 (*COI*) gene [18]. The presence of a 600-bp band visualized by electrophoresis on a 1% agarose gel confirmed that the extracted DNA was of appropriate PCR amplifiable quality.

For the molecular detection of *Leishmania* spp. in the tissues (ears and spleens) of the Norway rat, total genomic DNA was extracted from approximately 150 mg of ear and 100 mg of spleen samples using the QIAamp Blood and Tissue Kit (QIAGEN, Hilden, Germany), according to the manufacturer’s instructions. Each DNA sample was eluted in 200 μl of nuclease-free water and frozen at − 20 °C until use. The concentration and quality of the DNA obtained from these tissues were determined on a NanoDrop OneC spectrophotometer (Thermo Fisher Scientific). The primer pair ACTBF 5ʹ-TCCATCATGAAGTGTGACGT-3ʹ and ACTBR 5ʹGAGCAATGATCTTG ATCTTCAT-3ʹ was used to detect the beta-actin gene as a system control check; the presence of a 150-bp band confirmed that the extracted DNA was of appropriate PCR amplifiable quality [19].

*Leishmania* spp. DNA was detected by qPCR using the primers LEISH-1 (5-AACTTTTCTGGTCCTCCGGGTAG-3), LEISH-2 (5-ACCCCCAGTTTCCCGC C-3) and the TaqMan-MGB probe (FAM-5-AAAAATGGGTGCAGAAAT-3-non-fluorescent quencher-MGB) [20]. Each PCR reaction volume contained 1 µl of template DNA and a reaction master mix [2× SsoAdvanced Universal Probes Supermix (Bio-Rad Laboratories, Hercules, CA, USA), 0.9 µM of each primer and 0.2 µM of probe]. All qPCR assays were run with appropriate controls, including the non-template control (NTC) and positive control (DNA obtained from *L. major* Friedlin strain MHOM/IL/80/Friedlin; V1). Thermocycling conditions included an initial denaturation step of 4 min at 94 ºC, followed by 40 cycles of 95 °C for 15 s and 60 °C for 1 min.

The qPCR was carried out in the CFX96 real-time PCR detection system (Bio-Rad Laboratories). Threshold cycle (Ct) values were calculated automatically by CFX Manager 3.1 software (Bio-Rad Laboratories) using default parameters. The number of parasites was calculated using 123-bp fragments amplified by the LEISH 1 and LEISH 2 primers and based on the mass of the *Leishmania* genome (71.8 fg) and the number of copies of the histone H2B gene (1000 copies). Standard curves were constructed in triplicate by serial dilutions of genomic DNA from *L. infantum* promastigotes (reference strain: MHOM/BR/1974/PP75) with the dilution factor increasing by 10 to 10^–7^ (1.10 × 10^7^ parasites). The parasite number was determined by interpolating the mean of Ct) values of the samples in the standard curves.

Some results were repeated three more times for validation purposes. The mean and standard deviation of Ct values of each standard curve dilution were calculated using the GraphPad Instat software package (GraphPad Software Inc., San Diego, CA, USA).

*Leishmania* species were identified by amplifying the coding region of heat shock protein 70 (HSP70) using the 70-IR-D (5ʹ-CCAAGGTCGAGGAGGTCGAC TA-3ʹ) and 70-IR-M (5’-ACGGGTAGGGGGAGGAAAGA-3ʹ) primers [21]. The PCR products that generated amplification bands of approximately 700 bp were examined by electrophoresis in agarose gels and purified using NucleoSpin Gel and PCR Clean-up procedure (MACCHEREY-NAGEL, Düren, Germany), according to the manufacturer's instructions. DNA sequencing was carried out in the Genomics Unit IPBLN of López Neyra Institute, CSIC, Granada. DNA sequencing of the amplified product was submitted to the BLAST (NIH) database.

## Results and discussion

The selected sand fly females were identified as *Phlebotomus perniciosus,* the most abundant sand fly vector in Spain [8], which, in addition to *P. ariasi*, has been credited as a competent vector of *L. infantum* in the Iberian Peninsula [22]. The presence of these two main leishmaniasis vectors is known due to the monitoring of the city of Barcelona by ASPB (Agència de Salut Pública de Barcelona), the institution responsible for vector surveillance and control in the city [23].

The sequence of the 3'-untranslated region (UTR) is shown in Additional file 1: (Figure S1). The results of the NCBI BLAST analysis of this sequence verified that the amplicon corresponded to a non-transcribed region of the UTR of HSP70 (700 bp) corresponding to chromosome 28 of *Leishmania infantum*, with a 61% coverage and an E of 2.00E^−156^ and a percentage of identity of 89.86%. Similar values were obtained for *Leishmania chagasi*.

A phylogenetic tree was created with the same program (Additional file 2: Figure S2), showing how the amplified region corresponds to *L. infantum* /*L. chagasi* with a high identity.

*Leishmania infantum* DNA was detected in 14 out of the 27 (51.9%) sand fly individuals analyzed. The quantitative results obtained by the qPCR method for the infected *P. perniciosus* are shown in Table 1. Regarding parasite loads, sand flies captured in a leishmaniasis focus near Madrid (Spain) were classified into different groups [24]. According to our results, most of the sand flies (85.7%) presented moderate loads (between 10 and 1000 parasites) [24]. However, in the Madrid focus, the highest parasite load found, considered to be a very high load, was about 100,000 parasites. In our study, we detected an individual of *P. perniciosus* with a parasite load of > 1,500,000 and another one with an extraordinary parasite load of > 34,000,000 (Table 1) (Additional file 3: Figures S3, S4).

**Table 1:** Real-time PCR results for the 14 *Leishmania infantum*-positive *Phlebotomus perniciosus* individuals captured in the Barcelona underground sewer system

*P. perniciosus* ID numbers	qPCR results^a^	Estimated* n* of parasites	Standard deviation
	Ct		
1	32.10	157.18	17.88
2	31.62	220.37	40.32
3	30.81	371.93	24.20
4	32.35	134.98	32.22
5	29.38	979.45	65.34
6	33.97	65.57	28.05
7	18.31	1,528,989.74	446,327.06
8	13.84	34,005,842.84	6,927,767.90
9	32.10	157.18	17.88
10	31.62	220.37	40.32
11	30.81	371.93	24.20
12	32.35	134.98	32.22
13	29.38	979.45	65.34
14	33.97	65.57	28.06

Cut-off established for a positive result: Ct values < 36

^**a**^Values are the mean of the three replicates of each sample

*Leishmania infantum* infection was also investigated in 24 individuals of *R. norvegicus. L. infantum *was found to be present in 10 out of the 24 rats (41.7%) studied. The protozoan was found in the spleens of seven of the sampled rats (i.e. in 70% of the infected rats). *Leishmania infantum* was also found in the ears of four rats (i.e. 40% of the* Leishmania*-positive ones). Table 2 shows the respective qPCR quantitative results in *R. norvegicus* obtained in the spleens and ears of the* Leishmania*-positive individuals (Additional file 4: Figures S5, S6; Additional file 5: Figures S7, S8). One of the rats was determined to have an estimated load of > than 430,000 parasites per 150 mg ear tissue (*R. norvegicus * no. 8 in Table 2).

**Table 2:** Real Time PCR results for the spleens and ears of the 10 *Leishmania infantum*-positive *Rattus norvegicus* captured in the Barcelona sewer system

*Rattus norvegicus* ID numbers	qPCR results ^a^		
*R. norvegicus *spleens	Ct	Estimated* n* of parasites/100 mg	Standard deviation
1	35.45	6.71	2.34
2	35.24	8.43	2.22
3	28.06	794.89	74.66
4	35.24	8.43	2.22
5	35.07	29.43	3.49
6	23.10	20,156.21	1,987.96
7	30.06	445.04	38.93

*R. norvegicus *ears	Ct	Estimated* n* of parasites/150 mg tissue	Standard deviation
1	29.82	1222.35	242.37
8	18.09	430,119.63	86,890
9	34.87	10.66	2.32
10	33.86	84.33	37.63

Cut-off established for a positive result: Ct values < 36

^a^ Mean values of the three replicates of each sample

In six Norway rats the protozoan was present only in the spleen (rat ID numbers 2–7 in Table 2). One rat presented *Leishmania* in the spleen and the ear (rat number 1 in Table 2), and three only in the ears (rat numbers 8–10 in Table 2).

Despite the limitations of this study, the most important of which are the low number of sand flies/rats analyzed and the limited period of time in which the sampling was carried out, the results obtained are relevant. Taking into account both the high percentage of *L. infantum-*infected rats in the Barcelona underground sewer system [9] and the suitability of the sewers as a breeding site for sand flies [4], we expected a positive result for *L. infantum* in the phlebotomines studied, although not at such a high rate (51.9%).

Data on the prevalence of *L. infantum* in sand flies in urban areas of the Mediterranean basin are scarce. In Spain, entomological surveys carried out in four municipalities in the province of Madrid (considered a focus of human leishmaniasis) revealed a *P. perniciosus* prevalence of 8.9% over a 7-year period, with the highest infection rate, 15.2%, in Leganés, one of the towns included in the study [25]. Likewise, in the cities of Marseille (France) and Catania (Sicily, Italy), a prevalence of 5% and 11% *Leishmania* DNA was found in 72 and 99 *P. perniciosus*, respectively [26, 27]. A prevalence of 58.5% was found in *P. perniciosus* in a peri-urban area of Madrid, related to the human leishmaniasis focus mentioned above [28].

Most of the remaining data on *L. infantum* prevalences in *Phlebotomus* spp. in Spain were obtained in rural areas. For example, 12 out of 31 *P. perniciosus* (38.7%) were found to be infected by *L. infantum* in a study conducted in Catalonia [29]. A prevalence of 12.5% (8 positive individuals from 64 *P. perniciosus* analyzed) was found in a recent study in south-eastern Spain [30]. However, these values are notably higher (up to 58.5%) when studies focus on areas of *Leishmania* outbreaks [22]. Prevalences found in rural areas of other countries of the Mediterranean basin are low, varying between 0.1% and 15% [31–33]. The prevalences are also higher and vary between 37.6% and 60.5% in high endemic areas of leishmaniasis in Tunisia and Italy [34–36].

The high prevalence of *L. infantum* found in the sand flies from the underground Barcelona sewers in our study is consistent with those found in active leishmaniasis foci in the Mediterranean basin.

Regarding the quantitative results, to our knowledge, the highest *L. infantum* parasite load detected in previous studies in naturally infected sand flies was 2,820,246 ± 106,072 in an individual of *Lutzomyia longipalpis* in Brazil [37]. We detected a load of 1,528,989.74 ± 446,327.06 in a sand fly (number 7, Table 1) and an astounding parasite load of 34,005,842.84 ± 6,927,767.90 in another *P. perniciosus* individual (number 8, Table 1).

Regarding the results obtained in the rats studied, although the leishmaniasis prevalence was higher than that obtained in our previous study, the difference is not statistically significant (41.7 vs. 33.3%;* χ*^2^ = 0.262,* df* = 1, *P* = 0.4748). It is worth mentioning that, in addition to the spleens, *L. infantum* was also detected in the ears of 40% of the infected rats. One of the rats studied harbored a load of 3,089,047 ± 579,268 parasites/g ear tissue (number 8, Table 2). Ear skin, in addition to blood, is directly accessible to sand flies, which preferentially feed on certain body parts, such as the ears and nose [38]. An experimental infection revealed persistent parasite depots at bite sites in dog skins [38]. The large number of parasites we found in the ear of the rat probably represents one of these depots in a naturally infected *R. norvegicus*.

 CanL has always been considered to be a public health problem in endemic countries based on evidence that cases of CanL are known to precede human cases of the disease, and it is well known that dogs act as a reservoir [3, 4]. Therefore, leishmaniasis in dogs undoubtedly plays an important role in the maintenance of transmission levels and the spread of the disease, specifically in urban areas [39, 40]. In fact, culling dogs, although controversial, seems to be the only effective reservoir control method available to date [41]. However, the control measures taken specifically in urban areas that have involved the euthanasia of seropositive dogs [39, 42] did not modify the incidence of leishmaniasis in humans and dogs, suggesting the possible involvement of other reservoirs of infection [43]. Regarding sand flies, urban environments in Spain had previously not been considered suitable for *P. perniciosus* [22]. Our studies clearly demonstrate the importance of the trapping site for finding a large number of *Leishmania*-infected sand flies and rats. To our knowledge, the present study is the first and only study to date to assess the presence of *L. infantum* in rats living in underground sewage systems of urban areas, where intense contacts between rat and sand fly individuals have apparently led to a high prevalence of leishmaniasis, in turn potentiated by the presence of heavy parasite loads in certain hosts.

The identification of *L. infantum* in naturally infected sand flies is important for predicting the risk and expansion of leishmaniasis in endemic areas [29]. The high prevalence of natural *L. infantum* infection in a small sample of *P. perniciosus* trapped in sewers, as detailed in the present study, represents a clear risk of transmission, not only to dogs, but also to the human population. It is important to note that sand flies can find suitable ways to move from sewers to urban surfaces, since they are able to use open structures that connect both types of environments, such as catch basins or storm drains, among others. Once sand flies arrive at the surface, they can also bite humans/dogs close to urban green areas, as opportunistic blood feeding patterns have been demonstrated in *P. perniciosus*, including anthropophilic behavior [25].

Miró and López-Vélez [5] predicted in 2018 that “In the near future, we are likely to find new animal reservoirs to which special attention will have to be paid as these could even further complicate the epidemiological situation of human and animal leishmaniosis”. Recent epidemiological studies of leishmaniasis carried out in the city of Barcelona showed that autochthonous transmission occurs and is even on the increase [4, 44]. According to the results of the present study, the Norway rat *R. norvegicus* is likely to be this new reservoir, as we already indicated in our previous study [9], and, in addition to dogs, could be sustaining leishmaniasis levels in urban areas.

Sewer rat populations are important to humans since they could act both as a reservoir of individuals to re-colonize the surface after successful control actions and as reservoirs, not only of leishmaniasis but also of other parasitic zoonoses [10, 45, 46]. In fact, the rat harboring the highest *L. infantum* load was also infected by three other parasitic zoonotic agents (*Hymenolepis nana, H. diminuta* and *Taenia taeniaeformis larvae*) (unpublished data).

The obvious complexity of *L. infantum* control in rats represents a challenge, particularly in the COVID-19 scenario in which, in addition to a disruption in the leishmaniasis control programs [47, 48], an increase in rat populations has been observed in several cities due to lockdowns and the interruption of control measures [49, 50].

## Conclusions

This is the first report of high molecular levels of leishmaniasis in sand flies captured in urban sewers and the first time *L. infantum* has been detected, in addition to in the spleen, also in the ears of infected rats captured in the same underground sewer system of the city of Barcelona. We found, among both sand flies and rats, high parasite loads in certain individuals. As a result, Norway rats, in addition to dogs, are likely to act as reservoirs of leishmaniasis in cities where the sewer systems seem to offer the ideal scenario for leishmaniasis transmission. Therefore, to achieve the WHO 2030 target on the elimination of leishmaniasis as a public health problem successfully, an efficient control strategy against leishmaniasis in rats and sand flies should be implemented, particularly in the sewer systems of urban areas of endemic countries.

## Supplementary Information

**Additional file 1**: Sequence of the 3'-UTR region. **Figure S1**. The 3'-UTR region of the HSP70 gene was amplified with primers 70-IR-D (5'- CCAAGGTCGAGGAGGAGGTCGAC TA-3') and 70-IR-M (5'-ACGGGTAGGGGGGGAGGAAAGA-3').

**Additional file 2**: Phylogenetic analysis. **Figure S2.** Phylogenetic tree using 3'-UTR sequences of the HSP70 gene.

**Additional file 3:** Sand flies. **Figure S3.** Standard curve of* L. infantum* DNA. Standard curve obtained from serial dilutions of* L. infantum* DNA (10^8^ to 10^1^ parasites). Each point was tested in triplicate. Slope = — 3.70;  efficacy = 98 %;* R*^2^ = 0.991. **Figure S4.** Amplification curves. The plot showing the dilution of DNA concentrations (8 to 8 × 10^-7^ ng).

**Additional file 4**: Rat spleens. **Figure S5**. Standard curve of* L. infantum *DNA. Standard curve obtained from a series of dilutions of* L. infantum *DNA (10^8^ to 10^1^ parasites). Each point was tested in triplicate. Slope = -3.367; efficacy = 98.2%;* R*^2^ = 0.998. **Figure S6**. Amplification curves. The plot showing the dilution of DNA concentrations (8–8 × 10^-7^ ng).

**Additional file** **5**: Rat ears. **Figure S7.** Standard curve of* L. infantum* DNA. Standard curve obtained from a series of dilutions of* L. infantum* DNA (10^8^ to 10^1^ parasites). Each point was tested in triplicate. Slope = -3.47; efficacy = 94.1%;* R*^2^ = 0.998. **Figure S8.** Amplification curves. The plot showing the dilution of DNA concentrations (8 to 8 × 10^-7^ ng).

## Data Availability

Materials and data supporting our findings and conclusions are included in the article. The GenBank accession number of the submitted nucleotide sequence is ON364134.
