# Supplementary material for: Molecular detection of Leishmania infantum in rats and sand flies in the urban sewers of Barcelona, Spain
Source: Parasit Vectors. 2022 Jun 16;15:211. doi: 10.1186/s13071-022-05309-4 (PMC9201797; doi:10.1186/s13071-022-05309-4)
Supplement: Supplementary file 1 — Additional file 1: Sequence of the 3'-UTR region. Figure S1. The 3'-UTR region of the HSP70 gene was amplified with primers 70-IR-D (5'- CCAAGGTCGAGGAGGAGGTCGAC TA-3') and 70-IR-M (5'-ACGGGTAGGGGGGGAGGAAAGA-3'). [file 13071_2022_5309_MOESM1_ESM.docx]

**Additional file 1: Sequence of the 3'-UTR region**

**Figure S1:** The 3'-UTR region of the HSP70 gene was amplified with primers 70-IR-D (5' - CCAAGGTCGAGGAGGAGGTCGAC TA-3') and 70-IR-M (5' - ACGGGTAGGGGGGGAGGAAAGA-3').

GGTTACCCATAGCCTGGGAAGATAATTAGTTGCTTGAGTGACATAAATTT

TTTTTCTTGAACTAAATATGGATCCTTTTTGAAAAAAATTGAACCTGATT

TTTTCCGATGCCCAACTTGGTTGCAAAGGCCCCCCCCTAAAGTAGACAAG

AAAAGGTATTTTGGCACAATGTGTGTAGATAATTAGAAAGCACACCTTAT

TGCGGAAAACATACGCGACAGAGACTTGAGAATCCCTCCCTTTTCACCCC

CCGATTCCTAGCAAATGCGAGCTGCCGGAGTGCGTGTGATGCGCGTGTGA

GCGCATCTGCTCGTTTGCGAGCGAGCATGGCGGGCGTGCGGCAGCGGCGG

CGTGTTGGCCGGTAGTGGTGGAGAGGCGGCGTGCTGGCAGCTGTGTGTGT

GTGTGTATGTGCGTGCGACCCACGTGTGCTCGGCGTGTAATGCCCGCACA

CACACTGCTGCGCAAGCACTGCCTCTATCGTTGGCCTCTGTGCTGCTGCT

CTTCCTCTACACTTCGCATGTGTGAGTGGGGGGGGGGGGGTGTCCCTCCC

CTGTGTGTGTGTGTGTGTGTGTAAATATGTGTGTGCCCCTCCCCCCCAAA

CTGCCCCCCCCCGGGAGAGAGAAAACACACACACCCCCTTGTGCTCTTTG

AGAAAAAGCGGTTGAGTAAAACTCTTTCTTTTTCCCAAAAAAAAAAAAAA

A
