# Supplementary material for: Molecular detection of Leishmania infantum in rats and sand flies in the urban sewers of Barcelona, Spain
Source: Parasit Vectors. 2022 Jun 16;15:211. doi: 10.1186/s13071-022-05309-4 (PMC9201797; doi:10.1186/s13071-022-05309-4)
Supplement: Supplementary file 2 — Additional file 2: Phylogenetic analysis. Figure S2. Phylogenetic tree using 3'-UTR sequences of the HSP70 gene. [file 13071_2022_5309_MOESM2_ESM.docx]

**Additional file 2: Phylogenetic Analysis**

**Figure S2:** Phylogenetic tree using 3'-UTR sequences of the HSP70 gene.
