# Supplementary material for: Molecular detection of Leishmania infantum in rats and sand flies in the urban sewers of Barcelona, Spain
Source: Parasit Vectors. 2022 Jun 16;15:211. doi: 10.1186/s13071-022-05309-4 (PMC9201797; doi:10.1186/s13071-022-05309-4)
Supplement: Supplementary file 3 — Additional file 3: Sand flies. Figure S3. Standard curve of L. infantum DNA. Standard curve obtained from serial dilutions of L. infantum DNA (108 to 101 parasites). Each point was tested in triplicate. Slope = — 3.70; efficacy = 98 %; R2 = 0.991. Figure S4. Amplification curves. The plot showing the dilution of DNA concentrations (8 to 8 × 10^-7^ ng). [file 13071_2022_5309_MOESM3_ESM.docx]

**Additional file 3: Sand flies**

**
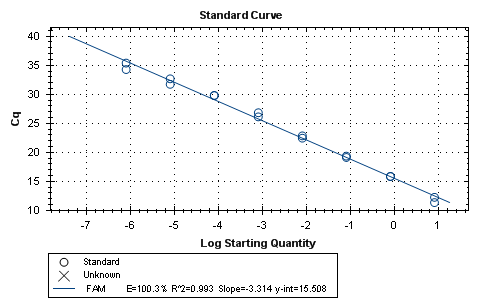
**

**Figure S3: Standard curve of *L. infantum* DNA. S**tandard curve obtained from serial dilutions of *L. infantum* DNA (10^8^ to 10 parasites). Each point was tested in triplicate. Slope = -3.70. Efficacy = 98 %; R2 = 0.991.

**
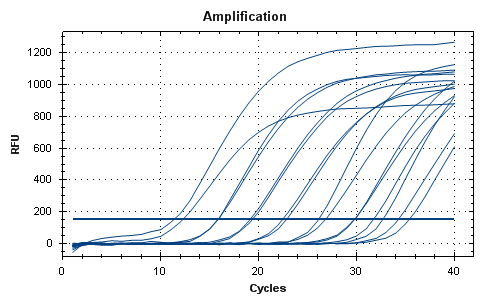
**

**Figure S4: Amplification curves.** The plot showing the dilution of DNA concentrations (8 to 8 x 10^-7^ ng).
