# Supplementary material for: Molecular detection of Leishmania infantum in rats and sand flies in the urban sewers of Barcelona, Spain
Source: Parasit Vectors. 2022 Jun 16;15:211. doi: 10.1186/s13071-022-05309-4 (PMC9201797; doi:10.1186/s13071-022-05309-4)
Supplement: Supplementary file 4 — Additional file 4: Rat spleens. Figure S5. Standard curve of L. infantum DNA. Standard curve obtained from a series of dilutions of L. infantum DNA (108 to 101 parasites). Each point was tested in triplicate. Slope = -3.367; efficacy = 98.2%; R2 = 0.998. Figure S6. Amplification curves. The plot showing the dilution of DNA concentrations (8–8 × 10-7 ng). [file 13071_2022_5309_MOESM4_ESM.docx]

**Additional file 4: Rat spleens**

**
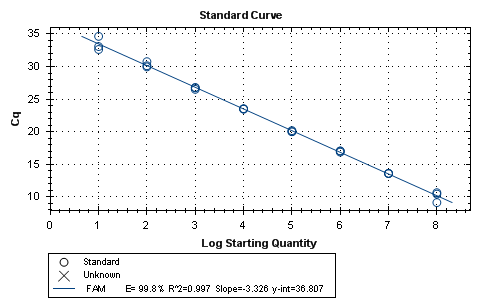
**

**Figure S5: Standard curve of *L. infantum*** **DNA**. Standard curve obtained from a series of dilutions of *L. infantum* DNA (10^8^ to 10^1^ parasites). Each point was tested in triplicate. Slope = -3.367; Efficacy = 98.2 %; R2 = 0.998.

**
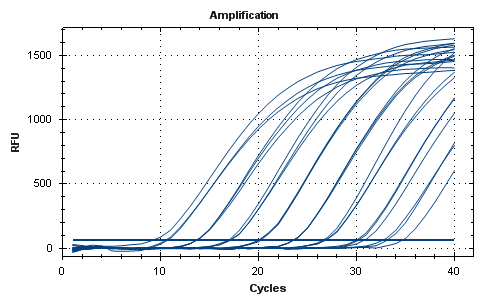
**

**Figure S6: Amplification curves.** The plot showing the dilution of DNA concentrations (8 to 8 x 10^-7^ ng).

**Additional file 5: Rat ears**

**
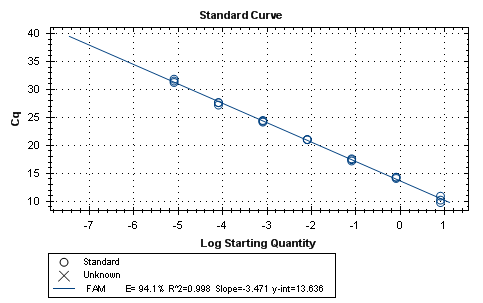
**

**Figure S7: Standard curve of *L. infantum* DNA. S**tandard curve obtained from a series of dilutions of *L. infantum* DNA (10^8^ to 10^1^ parasites). Each point was tested in triplicate. Slope = -3.47. Efficacy = 94.1; % R2 = 0.998.


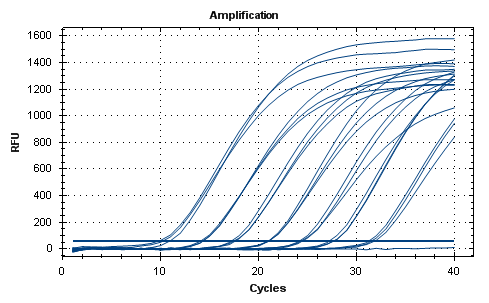


**Figure S8: Amplification curves.** The plot showing the dilution of DNA concentrations (8 to 8 x 10^-7^ ng).
